# Diagnosis, treatment and management of lipodystrophy: the physician perspective on the patient journey

**DOI:** 10.1186/s13023-024-03245-3

**Published:** 2024-07-11

**Authors:** Nivedita Patni, Craig Chard, David Araújo-Vilar, Helen Phillips, David A. Magee, Baris Akinci

**Affiliations:** 1 Division of Pediatric Endocrinology, Department of Pediatrics, UT Southwestern Medical Center, Dallas, TX USA; 2 Lumanity Inc., Great Suffolk Yard, 2nd Floor, 131 Great Suffolk Street, London, SE1 1PP United Kingdom; 3 UETeM-Molecular Pathology of Rare Diseases Group, Institute of Biomedical Research (CIMUS), School of Medicine, University of Santiago de Compostela, Santiago de Compostela, Spain; 4 Chiesi Global Rare Diseases, 45 Mespil Road, Dublin, Ireland; 5 Depark, Dokuz Eylul University & Izmir Biomedicine and Genome Center (IBG), Izmir, Turkey

**Keywords:** Diagnosis, Generalized lipodystrophy, Metreleptin, Partial lipodystrophy, Patient journey, Physician perspective, Quality of life

## Abstract

**Background:**

Lipodystrophy syndromes are a heterogeneous group of rare, life-limiting diseases characterized by a selective loss of adipose tissue and severe metabolic complications. There is a paucity of information describing the experiences and challenges faced by physicians who have seen and treated patients with lipodystrophy. This study aimed to provide a better understanding of the physician’s perspective regarding the patient journey in lipodystrophy, including diagnosis, the burden of disease, and treatment approaches.

**Methods:**

Thirty-three physicians from six countries who had seen or treated patients with lipodystrophy were interviewed using a semi-structured questionnaire. Interviews were transcribed, anonymized, and analyzed for themes and trends. Four main themes were developed: (1) the diagnostic journey in lipodystrophy including the disease features or ‘triggers’ that result in the onward referral of patients to specialist medical centers with experience in managing lipodystrophy; (2) the impact of lipodystrophy on patient quality of life (QoL); (3) the use of standard therapies and leptin replacement therapy (metreleptin) in lipodystrophy, and (4) barriers to metreleptin use.

**Results:**

Participants reported that, due to their rarity and phenotypic heterogeneity, lipodystrophy cases are frequently unrecognized, leading to delays in diagnosis and medical intervention. Early consultation with multidisciplinary specialist medical teams was recommended for suspected lipodystrophy cases. The development and progression of metabolic complications were identified as key triggers for the referral of patients to specialist centers for follow-up care. Participants emphasized the impact of lipodystrophy on patient QoL, including effects on mental health and self-image. Although participants routinely used standard medical therapies to treat specific metabolic complications associated with lipodystrophy, it was acknowledged that metreleptin was typically required in patients with congenital generalized lipodystrophy and in some acquired generalized and partial lipodystrophy cases. A lack of experience among some participants and restrictions to access remained as barriers to metreleptin use.

**Conclusions:**

To our knowledge, this is one of the first studies describing the qualitative experiences of physicians regarding the diagnosis and management of lipodystrophy. Other physician-centered studies may help increase the awareness of lipodystrophy among the wider medical community and support clinical approaches to this rare disease.

**Supplementary Information:**

The online version contains supplementary material available at 10.1186/s13023-024-03245-3.

## Introduction

Lipodystrophy syndromes represent a heterogeneous group of diseases characterized by a deficiency of adipose tissue affecting the whole body (generalized lipodystrophy, GL) or specific regions (partial lipodystrophy, PL) [1]. Lipodystrophy syndromes are rare, with an estimated global prevalence of 1.3–4.7 cases per million [2]. Generalized and partial forms of lipodystrophy may be genetic or acquired, giving rise to four main types: congenital generalized lipodystrophy (CGL), familial partial lipodystrophy (FPLD), acquired generalized lipodystrophy (AGL), and acquired partial lipodystrophy (APL) [1, 3]. CGL is defined by an almost complete lack of adipose tissue from birth or soon thereafter [1, 3, 4]. In FPLD, adipose tissue is distributed normally at birth, but is gradually lost from the upper and lower extremities, gluteal region and areas of the trunk, and typically becomes prominent around puberty in females and often later in males. Fat accumulation in FPLD may occur in the intra-abdominal region, face, neck, and mons pubis [3–5]. Adipose tissue loss in acquired forms of lipodystrophy can develop at any time in life but usually occurs during childhood or early adolescence [6–9]. In AGL, the onset of fat loss has been associated with the appearance of panniculitis, autoimmune diseases and anti-perilipin antibodies; however, many cases are idiopathic [1, 6, 7, 10–12]. The exact etiology of APL remains unclear, although several autoimmune diseases and various infections have been reported in some patients [4, 8, 9]. Adipose tissue loss in AGL is progressive and affects all areas of the body, while in a typical case of APL, fat loss is usually restricted to the face, arms, and upper body [1, 6, 10, 11]. Physical characteristics of lipodystrophy can include acromegaloid features in GL, Cushingoid features in PL, muscular appearance, and prominent subcutaneous veins [1, 4, 6, 10].

Adipose tissue loss in lipodystrophy causes ectopic accumulation of fat in organs such as the liver and muscles and can result in low levels of circulating leptin — a key adipokine regulator of satiety and energy homeostasis [3, 6, 13]. Consequently, patients with lipodystrophy can develop a range of complex metabolic complications (e.g., insulin resistance and diabetes mellitus, hypertriglyceridemia, non-alcoholic fatty liver disease, acanthosis nigricans), which can lead to organ damage [3, 6, 10, 14]. Major causes of mortality in lipodystrophy include liver, renal and cardiovascular disease, acute pancreatitis, and sepsis [3, 6, 10, 14]. Hyperphagia, due to leptin deficiency, frequently occurs in patients with lipodystrophy and can exacerbate comorbidities [3]. Reproductive dysfunction (e.g., polycystic ovary syndrome), psychological problems, fatigue, and pain (e.g., neuropathy, arthritis, and myopathy) have also been described within the broader disease symptomatology [15–18].

Treatment of lipodystrophy aims to improve or prevent long-term metabolic complications and organ damage. The standard clinical approach to lipodystrophy centers on lifestyle modification (i.e., low-fat diet and exercise if not contraindicated) and the use of glucose-lowering, lipid-lowering and cardiovascular medications to treat specific metabolic comorbidities [3, 6, 19]. Metreleptin, a 16 kDa recombinant analog of human leptin administered via subcutaneous injection, is the only medical therapy specifically approved for the treatment of the metabolic complications of lipodystrophy [20]. In the United States (US), metreleptin is approved as an adjunct to diet as replacement therapy to treat the complications of leptin deficiency in patients with GL [21]. In the European Union (EU), United Kingdom (UK), Canada and Brazil, metreleptin is approved as an adjunct to diet as replacement therapy to treat the complications of leptin deficiency in patients aged ≥ 2 years with confirmed GL, or in patients aged ≥ 12 years with confirmed PL for whom standard treatments have not achieved adequate metabolic control [22–25].

In patients with lipodystrophy, the application of appropriate medical treatment is dependent on the accurate and timely diagnosis of their condition. However, due to the rarity of lipodystrophy, cases are frequently unrecognized or misdiagnosed [3, 14]. Many patients receive a diagnosis only after they are referred to specialist medical centers with multidisciplinary clinical teams; however, the availability of these centers is limited in most countries [17, 20, 26, 27]. For some patients, severe comorbidities may have developed by the time a definitive diagnosis has been established [14, 28, 29]. In this regard, assessment of physicians’ perspectives regarding the challenges associated with diagnosing, treating, and managing lipodystrophy can provide new insights into the patient journey in lipodystrophy.

The objectives of this study were to capture the qualitative perspectives and experiences of physicians from the US and Europe who have either seen and subsequently referred patients with lipodystrophy to specialist medical centers or who have directly treated patients with lipodystrophy. Physicians were interviewed on their perceptions of: (1) the diagnostic journey of patients with lipodystrophy, (2) the impact of lipodystrophy on their patients’ quality of life (QoL); (3) the use of standard and lipodystrophy-specific therapies, including metreleptin; and (4) the barriers to metreleptin use. We discuss our findings in the context of previously published natural history studies and clinical investigations in lipodystrophy.

## Methods

### Study design and participants

This study comprised physician-based interviews conducted by Lumanity Inc. (previously Cello Health, https://lumanity.com/) between March and May 2021 on behalf of the study sponsor, Amryt Pharmaceuticals. To identify potential participants, a list of medical centers in the United States, France, Germany, Italy, Spain, and the United Kingdom that had a history of managing patients with lipodystrophy was prepared by the sponsor and used by researchers at Lumanity Inc. This list was not used exclusively — some participants were identified using snowball sampling techniques or were ‘free found’ from other medical centers. Physicians practicing adult and pediatric endocrinology, metabolic disease, diabetology, lipidology, and pediatric care were invited to participate in the study. A screening questionnaire prepared by Lumanity Inc. and approved by the study sponsor was used to validate participants before the start of the study. Screening questionnaires were completed by participants online or via telephone interview with a Lumanity Inc. researcher.

Participants were eligible for inclusion if they: (1) were based in a general hospital or private medical center and had either seen or treated patients with lipodystrophy, or if they had referred patients to a specialist center with experience in treating the disease; or (2) were based at a specialist center experienced in the treatment and management of lipodystrophy. All participants were required to have seen or treated an adult or pediatric patient with lipodystrophy within the 12 months before study initiation. Participants were not required to have used metreleptin for the treatment of lipodystrophy.

Participants were designated as key opinion leaders (KOLs) if they met one or more of the following criteria: (1) had been a session leader or chairperson for a national or international symposium or conference covering lipodystrophy (excluding local speaking engagements); (2) had presented at a national or regional symposium or conference on the subject of lipodystrophy in the last two years; (3) had written at least two major review articles discussing lipodystrophy in a peer-reviewed journal; (4) had written at least one textbook chapter or a full textbook on lipodystrophy and/or issues related to lipodystrophy within the last two years. KOL criteria were developed by Lumanity Inc. and approved by the study sponsor. All participants contributed voluntarily to the study after providing written consent and were offered an honorarium for their time. The identity of all participants was blinded to the study sponsor.

### Data collection

Interviews were conducted six weeks after completion of screening by researchers at Lumanity Inc. Interviews were overseen by one of the authors (CC), who has extensive experience in qualitative data collection for health service research. Before their interview, participants were notified that the information collected was confidential and that results would be reported as an aggregate or as anonymized quotations to the research sponsor. Participants were not provided with any materials before their interview. One-hour, web-enabled telephone interviews or virtual central location interviews were conducted for European and US-based participants, respectively. Only a researcher from Lumanity Inc. and the participating physician were present during the interviews.

An interview guide containing open-ended questions was used to prompt discussion and generate qualitative responses. Hypothetical lipodystrophy case studies were presented to the participants to support deeper discussion regarding the lipodystrophy types seen at their medical centers. These hypothetical cases contained information on the patient’s age at the time of their diagnosis, their current age, sex, medical history, and treatment regimens. Interviews conducted with participants from the US and Italy focused only on GL— this was in accordance with the approved label for metreleptin (US) and its restricted use in patients with PL presenting with severe metabolic and organ complications as per national guidelines (Italy). GL and PL were both discussed with participants from the other countries surveyed (France, Germany, Spain, and the UK). Notes were prepared by the researchers following the completion of each interview. No repeat interviews were conducted, and transcripts were not returned to participants.

### Data analysis

Data were collected during the interviews in the form of field notes and audio files. Following the interviews, researchers from Lumanity Inc. conducted debriefing sessions in which key points raised during the interviews were documented. Every effort was made to accurately capture and summarize each participant's views. Analysis was guided by the principles of researcher neutrality and systematic processes. Key trends were identified in each interview and compared with the findings of other interviews to generate themes and patterns on the collective experience in lipodystrophy.

Four main research themes were generated and explored based on the information received during interviews: (1) the diagnosis of lipodystrophy including the disease features or ‘triggers’ that result in the onward referral of patients to specialist care; (2) the impact of lipodystrophy on patient QoL; (3) the use of standard therapies and metreleptin in lipodystrophy; and (4) barriers to metreleptin use. Consolidated findings for each theme are presented here and are supported by anonymized quotations (Supplementary Information). Descriptive analyses included the number of patients and lipodystrophy types seen or treated by the participants, the medical specialty of the participant (including KOL status as determined here), the type of medical center in which the participants were based, and their history of using metreleptin.

## Results

### Participating physicians and patient numbers

Thirty-five physicians (10 from the US and five each from France, Germany, Italy, Spain, and the UK) were enrolled in this study. Participants were asked twice (at screening and during interview) for the number of patients with lipodystrophy who have been under their care. For the analysis presented here, the patient numbers provided during the interviews were used.

Initially, a total of 342 patients were reported during the interviews and comprised the number of current and historic cases seen or treated by the participants. Further inspection of these data, together with a second-round review of the audio recording transcripts, revealed that one participant from Spain had included localized lipodystrophy cases in their APL cohort. Another participant from the US reported having seen four patients during screening and 31 patients during the interview — the reasons for the marked difference between the screening and interview processes were not provided. Both participants were based in general, non-specialist medical centers and were excluded from the final analysis set. One other participant from the US, who was based in a specialist center with experience in treating lipodystrophy, reported that they had treated 23 patients (CGL, *n*=2; AGL, *n*=5; FPLD, *n*=11; APL, *n*=5) during screening but did not provide patient numbers during the interview. For this participant only, the number of patients provided during screening was used in the final analysis set.

**Fig. 1:**
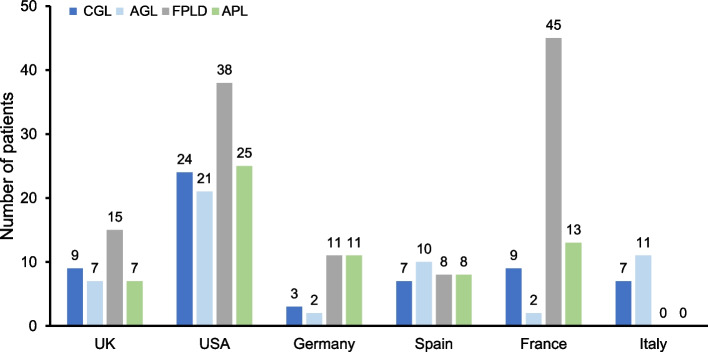
Distribution of the lipodystrophy types managed by the participating physicians. Legend: AGL, acquired generalized lipodystrophy; APL, acquired partial lipodystrophy; CGL, congenital generalized lipodystrophy; FPLD, familial partial lipodystrophy; UK, United Kingdom; USA, United States of America

Following these quality control and data refinement steps, the final analysis set comprised 33 participants and 293 patients (CGL, *n*=59, 20%; AGL, *n*=53, 18%; FPLD, *n*=117, 40%; and APL, *n*=64, 22%) (Fig. 1). It should be noted that although participants from the US provided the numbers of patients with PL currently or previously under their care, interviews focused only on their views and experience regarding the treatment and management of patients with GL, as per the interview criteria established in this study.

Eighty-five percent (*n*=28/33) of participants in the final analysis set were either endocrinologists (*n*=25) or pediatric endocrinologists (*n*=3). The remaining participants comprised three lipidologists (9%), one pediatrician (3%) and one diabetologist (3%) (Table 1). Fourteen participants (42%; four from Italy, three each from Germany and the US, and two each from France and the UK) were based in specialist treatment centers with experience in lipodystrophy; the other 19 participants (58%) were based in general hospitals or private medical centers. Twelve participants (36%) were considered KOLs (three from the US, two each from France, Germany, Spain, and the UK, and one from Italy) based on the criteria used in this study. Fifteen participants (45%, five from the US, three each from France and Italy, two from Germany, and one each from Spain and the UK) stated that they were using or had previously used metreleptin to treat lipodystrophy. Participants from the US and Italy who had experience with metreleptin were interviewed only on its use in GL in accordance with the local approved label and national guidelines, and the interview criteria used in this study.

**Table 1:** The medical specialty of the participants (final analysis set) in the study

**Medical specialty**	**USA (*n*=9)**	**France (*n*=5)**	**Germany (*n*=5)**	**Italy (*n*=5)**	**Spain (*n*=4)**	**UK (*n*=5)**	**Total (*n*=33)**
Pediatric endocrinologist	1			1	1 (1)		3 (1)
Endocrinologists	8 (3)	5 (2)	4 (2)	3 (1)	3 (1)	2 (1)	25 (10)
Lipidologist						3 (1)	3 (1)
Diabetologist			1				1
Pediatrician				1			1
Total	9 (3)	5 (2)	5 (2)	5 (1)	4 (2)	5 (2)	33 (12)

The number of KOLs for each country is shown in parentheses

*KOL* Key opinion leader

### Participant perspectives on the diagnostic journey of patients with lipodystrophy

There was agreement among the participants that, due to the rarity and phenotypic heterogeneity of lipodystrophy, patients can experience long delays in diagnosis or be misdiagnosed. They stated that CGL cases were more likely to be diagnosed at a younger age compared with other lipodystrophy types due to the generalized onset of fat loss and development of comorbidities during childhood. In contrast, they reported that, in their experience, AGL and PL cases usually do not receive a diagnosis until at least adolescence, when changes in fat distribution become apparent and metabolic complications worsen. It was further remarked that for some patients, severe comorbidities may have developed by the time a definitive diagnosis has been established. To illustrate this, participants from specialist centers described patients who were referred to them following inadequate responses to long-term treatment for diabetes and hyperlipidemia and who were subsequently diagnosed with lipodystrophy once under the care of their team.

### Participant perspectives on the impact of lipodystrophy on patient QoL

Participants recognized that lipodystrophy has a significant impact on their patients’ QoL. The primary QoL concerns of their adolescent and young adult patients centered on their physical appearance and emotional well-being. Patients often struggle with their body image and their metabolic condition, while some patients with PL can blame themselves for their regional fat accumulation. Participants reported that some of their patients seek assistance from mental health professionals to help improve their well-being. The long-term QoL concerns of patients included the impact of disease comorbidities on their life expectancy and their ability to have a family, especially for female patients.

The need for life-long medical care in lipodystrophy also emerged during interviews including the transition of patients from pediatric to adult clinical teams, which can be a source of anxiety for patients and their families. Participants noted that this transition of care generally occurs around 16-18 years of age and often coincides with the worsening of metabolic complications.

### Participant perspective on standard therapeutic approaches to lipodystrophy and the use of metreleptin

**Fig. 2:**
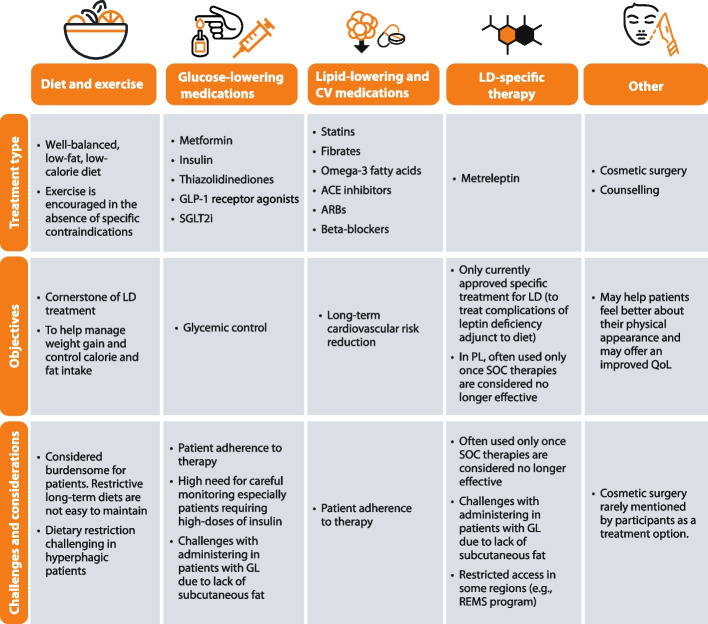
Summary of the participants’ views on standard and disease-specific therapies for lipodystrophy. Legend**:** ACE, angiotensin-converting enzyme; ARB, angiotensin receptor blockers; CV, cardiovascular; GLP-1, glucagon-like peptide 1; LD, lipodystrophy; QoL, quality of life; REMS, risk evaluation and mitigation strategy; SGLT2i, sodium/glucose cotransporter-2 inhibitors

Participants encouraged their patients to adopt a balanced, low-fat diet, to avoid alcohol and smoking, and to exercise frequently (unless contraindicated). They also reported using a broad range of standard therapies (e.g., glucose- and lipid-lowering agents, and cardiovascular medications) directed towards the metabolic complications presented by the patient. Participants reported that the limited efficacy of standard therapies resulted in the use of metreleptin in many patients, especially patients with CGL and those patients with AGL and PL who presented with severe metabolic complications and/or organ system complications. None of the participants reported using metabolic or cosmetic surgery in their patients with lipodystrophy. The participants’ views on the treatment options for lipodystrophy are summarized in Fig. 2.

### Participant perspective on the barriers to metreleptin therapy for lipodystrophy

Several barriers to metreleptin therapy were discussed during the interviews including restricted access to and use of metreleptin in some countries. Due to the reported risk of anti-metreleptin antibodies and lymphoma during therapy, metreleptin is available only through a restricted program under a risk evaluation and mitigation strategy (REMS) in the US. This program requires physicians to receive specific training before they can prescribe metreleptin. It was commented that enrollment in this program involves substantial administrative time and effort and is difficult to navigate. European participants observed that criteria from national guidelines determine patient eligibility for metreleptin therapy, especially in PL. Furthermore, prescription of metreleptin may be limited to a small number of designated national specialist centers (e.g., the National Severe Insulin Resistance Service located at Addenbrookes Hospital in the UK), requiring patients to travel long distances for medical consultations. Of note, several participants expressed frustration in losing connection with their patients after referral to specialist centers.

The willingness of patients to accept a daily, self-administered injection was perceived as another barrier to metreleptin use. Participants who had prescribed metreleptin stated that they spent considerable time encouraging and supporting their patients to adopt an injectable therapy as part of their treatment regimen. It was further observed that the treatment burden in lipodystrophy is high, with many patients disliking injectable therapies while some patients can tire of taking multiple concomitant medications leading to poor treatment adherence. Limited knowledge regarding the clinical efficacy and safety of metreleptin in lipodystrophy among some participants, as well as the challenges associated with country-level reimbursement and medical insurance coverage for patients, were also reported barriers to its use.

## Discussion

To our knowledge, this is one of the first interview-based studies exploring the perspectives of an international cohort of physicians regarding the management of lipodystrophy. Participants highlighted several challenges that they and their patients face, including long diagnostic journeys, impaired patient QoL, limited efficacy of standard therapies, and potential barriers to the use of metreleptin.

### Participant perspectives on the patient diagnostic journey in lipodystrophy

In this study, early diagnosis was regarded as a key step in the management of lipodystrophy, with physical appearance as a main driver of diagnosis. CGL cases were more likely to be younger at the time of diagnosis versus other lipodystrophy types, reflecting the earlier age at which the onset of fat loss occurs [1, 10, 28, 30–32]. A systematic literature review involving 1141 patients from 351 studies reported that the mean age at fat loss onset for CGL (0.3 years) was markedly lower compared with AGL (5.0 years), FPLD (9.9 years) and APL (8.2 years) [10]. More recently, a study involving 140 patients from Spain revealed that the phenotypic onset of lipodystrophy occurred during childhood in 95.6% of patients with GL (47.8% at birth; mean age 4.7 years), and during adolescence or adulthood in 91.7% of patients with PL (mean age 16.5 years) [30]. The findings of an international chart review study of 230 patients from the USA, Brazil, and Turkey also documented the earlier diagnosis of GL (mean 12.3 years) compared with PL (mean 33.7 years) [28].

The long patient journey to diagnosis was regarded as a key challenge in the management of lipodystrophy and is supported by observations from natural history and case studies of the disease. For example, in the international chart review study, the first symptoms of lipodystrophy were observed at a mean age of 9.2 years in GL and 24.7 years in PL; however, on average, diagnosis took an additional 3.1 and 9.0 years, respectively [28]. Similarly, data from Spain estimated mean diagnostic delays of 7.4 years for GL and 23.8 years for PL [30]. Differences in diagnosis times have also been seen between GL types. Analysis of a Turkish GL cohort involving 72 patients showed that in patients with CGL, the mean age for first clinic admission for symptoms was 11 years, with a mean delay between first clinic admission and diagnosis of 50 months. For AGL, the first clinic admission occurred, on average, at 22 years of age, with a delay between clinic admission and diagnosis of 86 months [31].

Many participants confirmed that metabolic disease may have developed in patients by the time a diagnosis of lipodystrophy has been established, thus highlighting the importance of prompt diagnosis and management of patients. In support of this, analysis of data from the overall Turkish GL cohort showed that the mean age of hypertriglyceridemia and hepatic steatosis development was 13 years — an average of 3 years before a diagnosis of lipodystrophy was confirmed (mean age at time of diagnosis was 16 years) [31]. In our study, the development and worsening of comorbidities were regarded as triggers for the referral of patients to specialist centers for lipodystrophy [17, 20, 26, 27, 31]. Early consultation with specialist medical teams for suspected cases of lipodystrophy offers the potential to expedite diagnosis and help guide appropriate medical intervention to optimize patient outcomes [31, 33].

Several factors may contribute to the long diagnostic journey in lipodystrophy. Diagnosis of lipodystrophy is based on clinical examination of the patient; however, precise diagnostic criteria have not yet been established [3]. As such, diagnosis largely depends on the healthcare professional’s experience with the disease, which in some instances may be limited [14, 17, 34, 35]. The phenotypic heterogeneity of lipodystrophy, especially in PL, can further confound diagnosis, as many clinical characteristics overlap with normal variation (e.g., low body fat levels and musculature in male patients with PL, leptin levels in PL) or other disease phenotypes (e.g., fat accumulation on the face and neck resembling Cushing’s disease in PL) [3, 14, 34]. The PL phenotype can also vary according to sex, with males often presenting with milder metabolic profiles compared with females [5, 36, 37].

### Participant perspective on the impact of lipodystrophy on patient QoL

All participants discussed how lipodystrophy impairs their patients’ QoL, including negative body image and low self-esteem. Observations from the LD Lync registry showed that patients with lipodystrophy had low scores for emotional well-being, social functioning, pain level, and general health [38]. A previous patient-oriented survey involving 10 patients with lipodystrophy and five of their caregivers reported that the symptoms frequently affecting patients include an inability to perform usual activities or attend work/school, impaired mobility, altered physical appearance, anxiety, depression, and pain or discomfort [17]. A recent study showed a significant impact of lipodystrophy on QoL and psychoemotional well-being [39]. This study also revealed that psychiatric disorders were underdiagnosed in many patients with lipodystrophy [39]. As with our study, earlier surveys also showed that female patients were concerned with the impact of lipodystrophy on their reproductive health [17].

### Participant perspective on standard therapeutic approaches to lipodystrophy and the use of metreleptin therapy

Many participants recognized that some patients with lipodystrophy require metreleptin to treat their metabolic complications. Clinical studies have demonstrated the sustained efficacy of metreleptin in lipodystrophy, characterized by improvements in insulin resistance, glycemic parameters, lipid abnormalities, and hepatic fat accumulation [32, 40–43]. It has been recently suggested that the metabolic effects of metreleptin start to develop within the first 48 hours following administration [44].

There was consensus among our participants that standard therapies have limited efficacy in CGL and that metreleptin is generally needed to limit disease progression in these patients. Notably, for AGL, some participants in our study stated that they reserve the use of metreleptin for patients presenting with severe comorbidities. This view contrasts with the current multi-society guidelines for lipodystrophy, which recommend metreleptin (with diet) as a first-line treatment for both CGL and AGL, including pediatric patients [3]. Data from the US National Institute of Health (NIH) metreleptin study has revealed a significant burden of disease in AGL in which the mean baseline HbA1c and fasting triglycerides levels were numerically higher in AGL (9.2% and 22.9 mmol/L) versus CGL (8.3% and 10.9 mmol/L) [42]. Furthermore, results from this study provided evidence for the efficacy of metreleptin in AGL, whereby the mean reductions from baseline to month 12 for HbA1c and fasting triglycerides were numerically greater for AGL versus CGL (AGL, –2.9% and –53.5%; CGL, –1.8% and –22.2%) [42]. Additional case studies have shown the effectiveness of metreleptin in AGL in real-world settings [45, 46].

Some participants noted that metabolic disease can be less severe in PL than GL and this may affect prescription practices. Although the metabolic disease burden is variable among patients with PL, it can be substantial in a subset of patients [10]. The efficacy of metreleptin has been demonstrated in PL, especially those with severe baseline metabolic complications [43, 47, 48]. In the NIH metreleptin PL study, a subgroup of patients who had baseline HbA1c ≥ 6.5% and/or triglycerides ≥ 5.65 mmol/L showed greater reductions in HbA1c (−0.9%), fasting triglycerides (−37.4%) and fasting plasma glucose (−1.9 mmol/L) versus the overall PL cohort (HbA1c −0.6%; fasting triglycerides, −20.8%; fasting plasma glucose, −1.2 mmol/L) following 12 months of therapy [43]. Real-world data from the French National Lipodystrophy Reference Network also showed that 89% (*n*=8/9) of patients with PL who had baseline HbA1c levels > 8%, triglyceride levels > 5.6 mmol/L and serum leptin levels < 4 ng/ml responded to metreleptin with respect to glucose homeostasis or triglyceride levels compared with 61% (*n*=11/18) from the overall PL population [48].

The use of metabolic surgery to improve metabolic control has been reported in a limited number of patients with partial lipodystrophy while cosmetic procedures may also improve patient well-being [14, 49–52]. Metabolic surgery is not recommended in patients with GL or severe lipoatrophy [29]. In our study, none of the participants reported using surgical interventions in their patients with lipodystrophy. However, as our interview guide did not include a structured question designed to collect data on surgical procedures performed in lipodystrophy, there may be an underreporting of such procedures in our data set. The high economic costs of these procedures, coupled with the limited data regarding their effectiveness and durability, especially in PL, may limit their use in clinical practice [29]. Furthermore, surgical interventions are not always reimbursed by healthcare systems, depending on the geographic location [52, 53].

### Participant perspective on the barriers to metreleptin therapy for lipodystrophy

Although previous clinical studies have illustrated the clinical effect of metreleptin in lipodystrophy, it was not used by all the participants in this study. Several reasons were cited for this including a restriction of its use as per national guidelines (particularly for patients with PL), a lack of reimbursement in some countries, and potential access issues for patients without appropriate medical insurance.

In the US, metreleptin is available only through a REMS program and carries a black box warning related to the need to monitor for lymphomas and immunogenicity during therapy. In the EU and UK, metreleptin carries a black triangle, indicating that it is subject to additional monitoring and requires a risk management strategy where educational materials are shared with physicians and patients [21–23]. These requirements stem from T-cell lymphoma cases reported in three patients with AGL following metreleptin therapy [54]. Two of these cases had immunodeficiency and hematological abnormalities prior to metreleptin initiation, while a single case of anaplastic large-cell lymphoma was reported in a pediatric patient without hematological complications before treatment. The development of lymphomas has been detected in cases of AGL before metreleptin therapy, and in other patients not receiving metreleptin at all [54]. Consequently, a causal relationship between metreleptin therapy and the development and/or progression of lymphomas has not yet been established [21–23, 54]. Nevertheless, these regulatory requirements may act as a barrier to the use of metreleptin.

No specific circumstances were highlighted as significant barriers to metreleptin use by participants who had access to and prior experience with this therapy. However, these participants acknowledged the considerable burden of treatment experienced by patients receiving a daily injectable therapy. Participants who had not previously used metreleptin cited a lack of familiarity and knowledge regarding its clinical efficacy and safety as barriers to its use but welcomed opportunities for further education on the therapy. Medical educational initiatives aimed at healthcare professionals and patients have been shown to support improved treatment strategies, effectiveness, and safety outcomes [55, 56].

### Study limitations

Our study has several limitations. First, data were collected as free-text using an interview guide that had not been pilot-tested before study initiation. Data were subsequently analyzed manually from which research themes were generated. This process may have resulted in a reporting bias or omission of details by the participants during the interview. Furthermore, our findings were not shared with participants for validation. As a validated questionnaire was not used, metrics or ratings regarding the responses given by participants were not recorded. Second, lipodystrophy is diagnosed clinically and relies on the physician’s experience with the disease. The degree of experience in recognizing lipodystrophy cases varied among the participants in our study. This was illustrated by the inclusion of localized lipodystrophy cases within the APL cohort of one participant (subsequently removed from the analysis). While every effort was made to accurately capture the number of patients seen or treated by the participants during interviews, it is possible that the final numbers provided by some participants may include localized lipodystrophy cases. Also, the patient numbers recorded during the interviews included current and historic cases, and it is plausible that, at a country level, some cases in the final analysis set may have been seen by more than one participant. This could lead to some duplicate data entries and artificial inflation of the final patient numbers reported. Third, some topics were not discussed or elaborated upon during the interviews, including the role of cascade screening to aid the diagnosis of potentially affected relatives of patients with inherited lipodystrophies and how differences between private and public healthcare settings may impact diagnosis and access to treatment, including metreleptin. Fourth, although the participants provided an overview of the reasons regarding the barriers to the use of metreleptin therapy, the data collected during the interviews did not permit a more robust analysis of these barriers. Finally, the participants in our study were based in countries where metreleptin was approved for use during the study period (between March and May 2021) and this may limit the generalizability of our findings.

## Conclusion

This study provides insight regarding the perceptions of physicians on the challenges in lipodystrophy. While further research is needed, physician-focused surveys such as this can support educational initiatives regarding lipodystrophy among the wider medical community. These initiatives may help improve diagnosis and referral pathways which are important steps for optimizing treatment and management in lipodystrophy.

### Supplementary Information

Supplementary Material 1.

## Data Availability

Data sharing is not applicable as no datasets were generated or analyzed during the current research. Interview transcripts are not publicly available and will not be shared by the corresponding author to protect participant identities.
