# Supplementary material for: Diagnosis, treatment and management of lipodystrophy: the physician perspective on the patient journey
Source: Orphanet J Rare Dis. 2024 Jul 11;19:263. doi: 10.1186/s13023-024-03245-3 (PMC11241872; doi:10.1186/s13023-024-03245-3)
Supplement: Supplementary file 1 — Supplementary Material 1. [file 13023_2024_3245_MOESM1_ESM.pdf]

Title: Diagnosis, treatment and management of lipodystrophy: the physician perspective on the patient journey (Supporting Information)

Nivedita Patni,[1] Craig Chard,[2] David Araújo-Vilar,[3] Helen Phillips,[4] David A. Magee,[4] Baris Akinci[5]

Affiliations:

[1]Division of Pediatric Endocrinology, Department of Pediatrics, UT Southwestern Medical Center, Dallas, TX, USA

[2]Lumanity Inc, Great Suffolk Yard, 2nd Floor, 131 Great Suffolk Street, London SE1 1PP, United Kingdom

[3]UETeM-Molecular Pathology of Rare Diseases Group, Institute of Biomedical Research (CIMUS), School of Medicine, University of Santiago de Compostela, Santiago de Compostela, Spain

[4]Chiesi Global Rare Diseases, 45 Mespil Road, Dublin, Ireland

[5]Depark, Dokuz Eylul University & Izmir Biomedicine and Genome Center (IBG), Izmir, Turkey

Anonymized, supporting quotations from participants

The supporting quotations presented here are used to illustrate the feedback received during the interview process.

1. Participant perspectives on the diagnosis of lipodystrophy

"………I think that when you have a patient with congenital generalized lipodystrophy (CGL), you're going to pick up on it pretty quickly…….... I think their parents will realize early on that is something wrong with their child." (Participant, USA)

"………It was very impactful to see a small girl (patient with CGL) with a complete lack of body fat all over the body. (Participant, Spain)

"………Since it is acquired [lipodystrophy], we might expect a later presentation." (Participant, Spain)

"………Diagnosis of acquired generalized lipodystrophy (AGL) is tougher because the phenotype is not as obvious, it occurs more gradually over time." (Participant, USA)

"………To get to the diagnosis is quite slow. I think this is a very rare condition, so I put it down as a very slow diagnosis. From the time you see [the patients] to the time the diagnosis is confirmed is a very slow process". (Participant, UK)

"………There is a lack of awareness among [general] healthcare professionals (HCPs) that prevents appropriate diagnosis". (Participant, France)

"………Some general practitioners know that there's something wrong, but they don't refer patients to specialists quickly enough, and that feels like wasted time for the patient…Because of these delays, the patient wasn't managed correctly, and that allowed the complications to progress". (Participant, France)

"………I think the initial problem is at the time of diagnosis, that is, when you don't know what is happening to the boy or the girl, there is a developmental delay, metabolic complications and as long as it is not diagnosed, all this period is a period of uncertainty, seeing many doctors, fears… once they are diagnosed, it is about managing the metabolic factors as best as you can". (Participant, Spain)

“………I think the frustration for me, is that it can take a long time for the diagnosis to be made. And I am very disappointed [not] to be referred sooner. I think when patients are struggling to get good control, why leave the patient for 5 years?” (Participant, UK)

“………The main issue is diagnosis, most of the patients we see have been found by chance because they attend surgeries for other conditions and their situation remained undiagnosed until then.” (Participant, Italy)

“……… [Patients] are frustrated and saddened that as it is a rare disease, patients may not meet the right HCP for a very long time and go undiagnosed or inadequately treated for years.” (Participant, Germany)

“………Patients often get referred directly to us in endocrinology, because they present with uncontrolled diabetes”. (Participant, France)

“………The transfer into adult care is a hard moment for all chronic patients, they know the pediatric hospital and doctors well, but they don’t know what is ahead of them.” (Participant, Italy)

“………She [patient with AGL] was registered at 15 which is considered a young age for diagnosis, treatment was challenging and led to complications. When you look back to tracking her record of the biochemical data… she had abnormal cholesterol and had pre-diabetes for probably more than 10-15 years, and she was on a statin and other treatment.

“What frustrates me, and the patients, is that it takes so long to get a proper diagnosis. Some of these people see several doctors, endocrinologists… sometimes, they’re told, ‘we don’t understand why you have diabetes; it doesn’t make sense’. Sometimes, they’re blamed due to their diet.” (Participant, USA)

“………Based on [the patient’s] underlying condition and insulin resistance profile, I am worried about their poor metabolic control and, honestly, during the last visit, she was very affected by the entire aesthetic situation”. (Participant, Spain)

2. Participant perspectives on the impact of lipodystrophy on patient quality of life (QoL)

"………These are patients that if we don't control them well, it is true that the complications, the endocrinologic and metabolic complications, can compromise not only their quality but also their quantity of life, that is, their life expectancy". (Participant, Spain)

"………My anticipation about my patient and the next ten years of her life being full of risk and potential disability". (Participant, USA)

"……… "Beyond aesthetics (which is what patients may worry about more), the condition causes a host of physical complications: diabetes and insulin resistance, high triglycerides, bone cysts, elevated transaminases, intolerance to cold temperatures, an elevated cardiovascular risk, risk of kidney damage" (Participant, Italy)

"………They find it hard to imagine themselves having a heart attack aged thirty, whereas I know that this is a real risk." (Participant, France)

"………Patients are very concerned about how aesthetic issues may affect their day-to-day life." (Participant, Italy)

"………lipodystrophy was honestly affecting the patient psychologically and we didn't have a lot of tools to improve the profile." (Participant, Spain)

"………Patients may struggle with social-psychological issues with some potentially requiring the help of psychologists / psychiatrists in addition to financial problems." (Participant, USA)

"………On average, [patients] they feel self-conscious, depressed and bit isolated." (Participant, Spain)

3. Participant perspective on standard therapeutic approaches to lipodystrophy and the use of metreleptin therapy

"………It's very important to control [patients] complications when they're young, because otherwise they're at a very high risk of developing complications as they age." (Participant, France)

“………[Regarding standard therapies]….We can’t completely protect them from metabolic or cardiovascular complications, and that makes their prognosis more complicated.” (Participant, France)

“………[Regarding metreleptin] I would feel confident, because it is getting to the heart of the disease, [I am] enthused that I have an option to present to the patient….and that we have valuable and specific treatments available.” (Participant, USA)

“………This type of treatment [metreleptin] makes a lot of physiological sense on paper; everything else is patching up problems that arise over time.” (Participant, Spain)

“………These are the [disease-specific] treatments that will be needed, but always building on a leptin base, because what fails in these patients is that their body doesn’t produce leptin because they don’t have any fat.” (Participant, Spain)

“………I personally have a bit more experience with the [congenital] generalized cases… I would probably initiate this generalized case a bit earlier on leptin because what I have seen in the literature…..I have seen more results on patients with generalized lipodystrophy (GL), so I would be more at ease to start them earlier on leptin… It is probably more what my knowledge is, and from my point of view that the generalized one is a more severe condition than the partial from that point of view.” (Participant, UK)

“………Metreleptin is available as a prescription, so at least, there’s some targeted treatment for these individuals” (Participant, USA)

“………I think understanding the data and understanding the potential side effects [of metreleptin], potential risks so that you can explain that to the patient and make a joint decision as to whether this is the right thing to do……….the patient has to be able to agree to the injections and understand any potential side effects and any deleterious effects, which they need to balance against their current position.” (Participant, UK)

4. Participant perspective on the barriers to metreleptin therapy for lipodystrophy

"………Metreleptin is tough to get because the paperwork for the Risk Evaluation and Mitigation Strategies (REMS) program is a pain and insurance companies may still reject it." (Participant, USA)

"………We have a lot of challenges getting patients on it here in the United States (US) and defining the diagnosis specifically enough to get approval" (Participant, USA)

"……… [metreleptin is brought in later] generally because of the difficulty of getting the treatment for the patient. When you request this medication, you really have to justify why they need it. You need to prove that you have done this and that and had genetic testing, or have done the 1st, 2nd, 3rd line treatment". (Participant, UK)

"………I think having more than one center, a few centers in the UK would potentially increase the chances of this therapy [being used]. Because now there is the question they have to travel a long, long, long distance to get the treatment, so that is why a lot of them will not go." (Participant, UK)

"………We did try very hard actually [to get this patient on metreleptin], but it wasn't an option…This person hadn't even travelled on a train, so to ask them to go across the country was really difficult" (Participant, UK)

"………What makes me frustrated is that I don't know how we will get the reimbursement approval for the treatment; we are fully dependent on the insurance company." (Participant, Germany)

"………we are dependent on the insurance's decisions – this could mean that the patient evolves additional complications if the insurance refuses." (Participant, Germany)

"………The issue is that I haven't used [metreleptin] yet, and whenever you haven't used something, you feel less confident with it." (Participant, France)

"………I need to know more about the drug and more experience with its use, I know it is a well-tolerated drug but I still lack experience." (Participant, Italy)

"………In order to engage the patient in overcoming the barriers to self-administration of a daily injectable, there has to be an engagement pathway there. You have to convince the patient that it is worth it and that they should stick with it, and in order for that to be true, patients generally have to have more advanced serious problems that they are trying to navigate through. Because otherwise they simply won't engage in this type of injectable therapy." (Participant, USA)

"………For the patients, it's particularly frustrating because they have to take a ton of medications, so then the chance of non-adherence is higher." (Participant, USA)

"……… [metreleptin] is an injection, and most patients would prefer to try to take oral treatments first, and only use injections if they're not controlled with oral medications." (Participant, France)

"………Before, they were just taking pills, but now we're asking them to take an injection, which is hard for them to accept when they don't have any symptoms." (Participant, France)
